# Antiviral activity of SAFER^®^, a commercial acidifying desiccant powder, against African swine fever virus

**DOI:** 10.3389/fvets.2024.1245569

**Published:** 2024-08-20

**Authors:** Thi Bich Ngoc Trinh, Elodie Lazenec, Thi Ngoc Ha Lai, Maria Matard-Mann, Luong Tan Phat, Anne Morvan, Anne-Cecile Delahaye, Pi Nyvall Collén, Thi Lan Nguyen, Van Phan Le

**Affiliations:** ^1^College of Veterinary Medicine, Vietnam National University of Agriculture (VNUA), Hanoi, Vietnam; ^2^Olmix SA, Bréhan, France

**Keywords:** ASFV, antiviral activity, body fluids, SAFER^®^, African swine fever

## Abstract

African swine fever (ASF) is one of the deadliest swine diseases, causing significant economic losses, threatening food security, and limiting pig production in affected countries. In the absence of an effective ASF vaccine, prevention and control of ASF depend mainly on effective biosecurity measures. In this study, the efficacy of SAFER^®^, a powdered disinfectant containing clay, an acid complex, and the active ingredient thyme essential oil, was tested against the ASF virus. The results showed that ASFV isolate (VNUA/HY/ASF1/Vietnam/2019) was inactivated by 3.5 and 5 Log_10_HAD_50_/ml after 20 and 120 min of treatment with SAFER^®^, respectively. When body fluids contaminated with ASFV, such as blood, saliva, urine, and feces, were treated with SAFER^®^ for 20 min, the ASFV titer was reduced by 1.6, 2.2, 2.0, and 2.2 Log_10_HAD_50_/ml, respectively.

## 1 Introduction

ASF was first described in the 1920s as an acute hemorrhagic fever disease that caused up to 100% mortality in domestic pigs in Kenya. ASF outbreaks have been reported in many countries on all continents, from Africa to Asia (1). The causative agent, ASFV, is a double-stranded DNA virus with a complex structure in tetrahedral morphology and a genome between 170 and 194 kbp with a virion diameter of 172–191 nm (2). It is a member of the genus *Asfivirus*, family Asfarviridae (3). Although ASFV is an enveloped virus, it is known to be stable under various conditions, such as the environment and animal products. There are many routes of transmission between uninfected and infected animals, either through direct contact between animals, bites from infected arthropods and soft ticks of the genus *Ornithodoros* (4), or indirect contact through contaminated fomites: feed, feed ingredients, pork products, or personnel (5). The virus can be found in all secretions and nasal fluids, which is a dangerous source of infection (6). Infected individuals’ blood, urine, and feces can be one of many potential sources of transmission in the environment, especially in domestic pigs. Previous studies have shown that these sources remain in the environment for weeks (7–10). Others showed that ASFV was very stable in swine manure from 11 to 160 days (8, 11) and depended on storage temperatures. Davies et al. (12) showed that the viral pathogen could survive between 8 and 15 days at 4°C in urine and fecal samples. Previous reports indicated that ASFV could survive for a long time in a protein-rich environment and could also survive a pH of 4–10 (13). The stability of ASFV is highly dependent on the matrix tested but is generally higher at low temperatures such as 4°C or −20°C, where infectious virus particles have been found in carcass samples after several months or even years of storage (5, 14–16). Effective thermal inactivation of ASFV was achieved at 56°C for 70 min or 60°C for 20 min (17).

The complexity of the viral genome and many other factors make the development of a safe and effective vaccine against ASF challenging (18). Therefore, decontamination practices should be the focus of disease prevention. Once an ASF outbreak is confirmed, eradication depends on applying a combination of measures to eliminate the pathogen. The critical role of cleaning is to remove dirt and soil that interfere with the effectiveness of disinfectants, as organic matter, especially proteins, prevents the inactivation of ASFV by disinfectants (19, 20). The World Organization for Animal Health (WOAH) has recommended using some chemical compounds or disinfectants to inactivate ASFV, e.g., 0.8% NaOH, 0.03–0.5% chlorine from hypochlorite, 0.3% formaldehyde, 3% ortho-phenylphenol with the 30-min contact time, and iodine. These disinfectants should inactivate approximately 4.0 Log_10_HAD_50_/ml of ASFV titers with a 30-min contact time. A previous report showed that ASFV titers in blood, rectal fluid, and nasal fluid were variable, ranging from 6 to 9 Log_10_HAD_50_/ml, 1 to 8 Log_10_HAD_50_/ml, and 1 to 4 Log_10_HAD_50_/ml, respectively (21). The lack of proper risk management for ASFV and the excessive ASFV load in the secretions and excretions of infected pigs eventually contaminate the culture medium or the environment, even after the application of disinfectants. Removing the virus from the environment and personal equipment is nearly impossible. Therefore, good on-farm biosecurity measures and staff training are needed to reduce the risk of virus spread. Using chemical disinfectants is a critical factor in reducing the incidence of the virus. However, due to their strong odor, toxicity, and carcinogenicity, they cannot be used in the presence of animals. Therefore, companies are looking for natural complementary solutions to chemical disinfectants to expand their activities and protect the environment in the presence of animals. This study aims to evaluate the efficacy of the commercial product SAFER^®^ in inactivating ASFV. To this end, the efficacy of SAFER^®^ was assessed for various ASFV concentrations in sterile water. Subsequently, tests were conducted with ASFV-contaminated body fluids such as blood, saliva, urine, and feces from contaminated animals to evaluate the product’s efficacy in organic media. Finally, an experimental design was established to evaluate the efficacy of SAFER^®^ on ASFV as a function of temperature, incubation time, and pH.

## 2 Materials and methods

### 2.1 Virus and disinfectants

For the experiment, the ASFV strain VNUA/HY-ASF1/Vietnam/2019 (8 Log_10_HAD_50_/ml), a highly pathogenic strain of the p72 genotype II (22), was propagated in porcine alveolar macrophage (PAM) cells. The PAM cells used in the study were collected from the lungs of healthy 8- to 10-week-old pigs purchased from a private farm in Hung Yen province and housed at the Animal Biosafety Research Facility of the College of Veterinary Medicine, Vietnam National University of Agriculture (VNUA). Viral titers, expressed as the amount of virus causing hemadsorption in 50% of infected cultures (HAD_50_/ml), were calculated according to the previously described method (23). The standard calibration curve was generated using cycle threshold (Ct) values obtained from serial 10-fold dilutions of known ASF virus titers in triplicate. In this study, all virus experiments were performed at the biosafety facility of the College of Veterinary Medicine, VNUA, Hanoi, Vietnam.

The product used in the present study is a commercial sanitizing product named SAFER^®^ (Table 1).

**Table 1:** Composition of product.

Disinfectant (Trade name)	Active ingredient
SAFER^®^	Clay Acidic complex Active principle of thyme essential oil

### 2.2 Calculation of viral load reduction

The reduction in viral load in response to SAFER^®^ was calculated as a Log_10_ value. The log reduction in the amount of virus recovered from the treated wells of the cell culture plate was calculated using the previously published formula (24):


$$\mathrm{Log}\;\mathrm{reduction}\;\left(\log_{10}\right)=T_{\mathrm{before}}-T_{\mathrm{after}}$$

where T_before_ (positive control) corresponds to the amount of virus recovered from the positive control wells without SAFER^®^ treatment, and T_after_ (treated) corresponds to the virus titer recovered from the SAFER^®^ treated wells.

### 2.3 Testing the efficacy of the antiviral activities of SAFER^®^

#### 2.3.1 Evaluation of the antiviral activity of SAFER^®^ at pH 3.2 against ASFV isolate at room temperature (RT) (25°C) in sterile water

The first experiment aims to validate the efficacy of SAFER^®^ in inhibiting the ASFV isolate (VNUA/HY-ASF1/Vietnam/2019), as determined by real-time PCR detection of viral genetic material, using different ASFV titers (10^3.5^, 10^5^, and 10^6.5^ HAD_50_/ml) in sterile water at ambient temperature (25°C). To achieve this, 0.3 g of SAFER^®^ powder with a pH of 3.2 was mixed with 1 mL of the virus solution containing various ASFV titers (10^3.5^, 10^5^, and 10^6.5^ HAD_50_/ml) in 12.56 cm^2^ Petri dishes and incubated at RT. At regular time points, the reaction of SAFER^®^ was stopped by adding 1 mL of neutralization broth (Neutralisant universal; http://www.indicia.fr), mixed well, and allowed to settle for 10 min at RT. No SAFER^®^ powder was added in the first step when preparing positive controls. Negative controls were prepared by replacing the ASF virus solution with 1 mL of H_2_O. Neutralization ability was confirmed by a control in which SAFER^®^ was mixed with neutralization broth before the addition of the ASF virus. Finally, the entire solution was collected in the Petri dish and centrifuged at 4000 rpm for 10 min. The supernatant was collected and used for DNA extraction (Supplementary Table S1). Each assay was performed in triplicate, and the results shown were the average of the real-time PCR values.

#### 2.3.2 Evaluation of the antiviral activity of SAFER^®^ at pH 3.2 against ASFV at RT (25°C) in contaminated fluids

Blood, saliva, urine, and fecal samples used in this study were diagnostic samples sent by the owners of family pig farms in Vietnam to the Key Laboratory of Veterinary Biotechnology at the College of Veterinary Medicine, VNUA, Hanoi, Vietnam, for ASF diagnosis. These samples were later confirmed to be ASFV-positive. Viral titers of these contaminated fluids were determined using Ct values from real-time PCR and converted to HAD_50_ values using a standard curve. The virus titers in blood, saliva, urine, feces, and spiked fecal solutions were 7.22, 4.10, 4.55, 2.88, and 5.0 Log_10_ HAD_50_/ml, respectively. Spiking feces artificially increased the viral concentration in feces by adding viral particles to the sample. This procedure was performed in the laboratory to obtain a high enough initial viral titer to evaluate the overall capacity of SAFER^®^ to reduce viral concentration in feces. The procedure for testing SAFER^®^ with contaminated blood, saliva, urine, feces, and spiking was the same as the protocol tested with the ASFV isolate (VNUA/HY-ASF1/Vietnam/2019) prepared in sterile water, except that the volume of neutralizing broth was increased from 1 mL to 2 mL to inactivate the SAFER^®^ product completely (Supplementary Table S2). Each assay was performed in triplicate, and the results presented were the average of 3 values obtained from real-time PCR.

#### 2.3.3 Effects of temperature, pH, and incubation time on the efficacy of SAFER^®^

The final test in this study addressed the effects of temperature, pH, and incubation time on the efficacy of SAFER^®^. For this purpose, an experimental design was designed to test ASFV reduction under different combinations of temperature (4°C, 25°C, and 35°C), incubation time (3, 7, 20, and 60 min), and pH (3.2, 3.7, and 4.2) at an initial viral concentration of 6.5 Log_10_HAD_50_/ml (Supplementary Table S3). For each of the 36 combinations, the reduction in ASFV titer was measured by real-time PCR.

### 2.4 DNA extraction and real-time PCR

DNA was extracted from contaminated blood, saliva, urine, and feces before and after SAFER^®^ treatment using the QIAamp DNA kit (Qiagen, United States) following the manufacturer’s instructions. ASFV detection was carried out using the VDx ASFV qPCR kit (Median Diagnostics, South Korea). In summary, 5 μL of the extracted DNA was combined with 10 μL of the 2X master mix and 5 μL of the 4X oligo mix in a PCR tube. The reaction was executed under the following conditions: 40 cycles of 95°C for 15 s and 58°C for 60s using a CFX96 Touch Real-Time PCR Detection System (Bio-Rad Laboratories Ltd., Hercules, CA, United States). Samples with a Ct value of less than 40 were considered positive for ASFV.

### 2.5 Statistical analysis

Each of the three parameters that may affect viral titer was measured at constant points: 4°C, 25°C, and 35°C for temperature; 3.2, 3.7, and 4.2 for pH; and 3, 7, 20, and 60 min. Each parameter was considered a factor (i.e., qualitative variable). Using R software (R Development Core Team), a 3-factor model ANOVA was applied with a risk of 5%.

## 3 Results

### 3.1 Antiviral activity of SAFER^®^ at pH 3.2 against ASFV prepared in sterile water at 25°C

**Figure 1:**
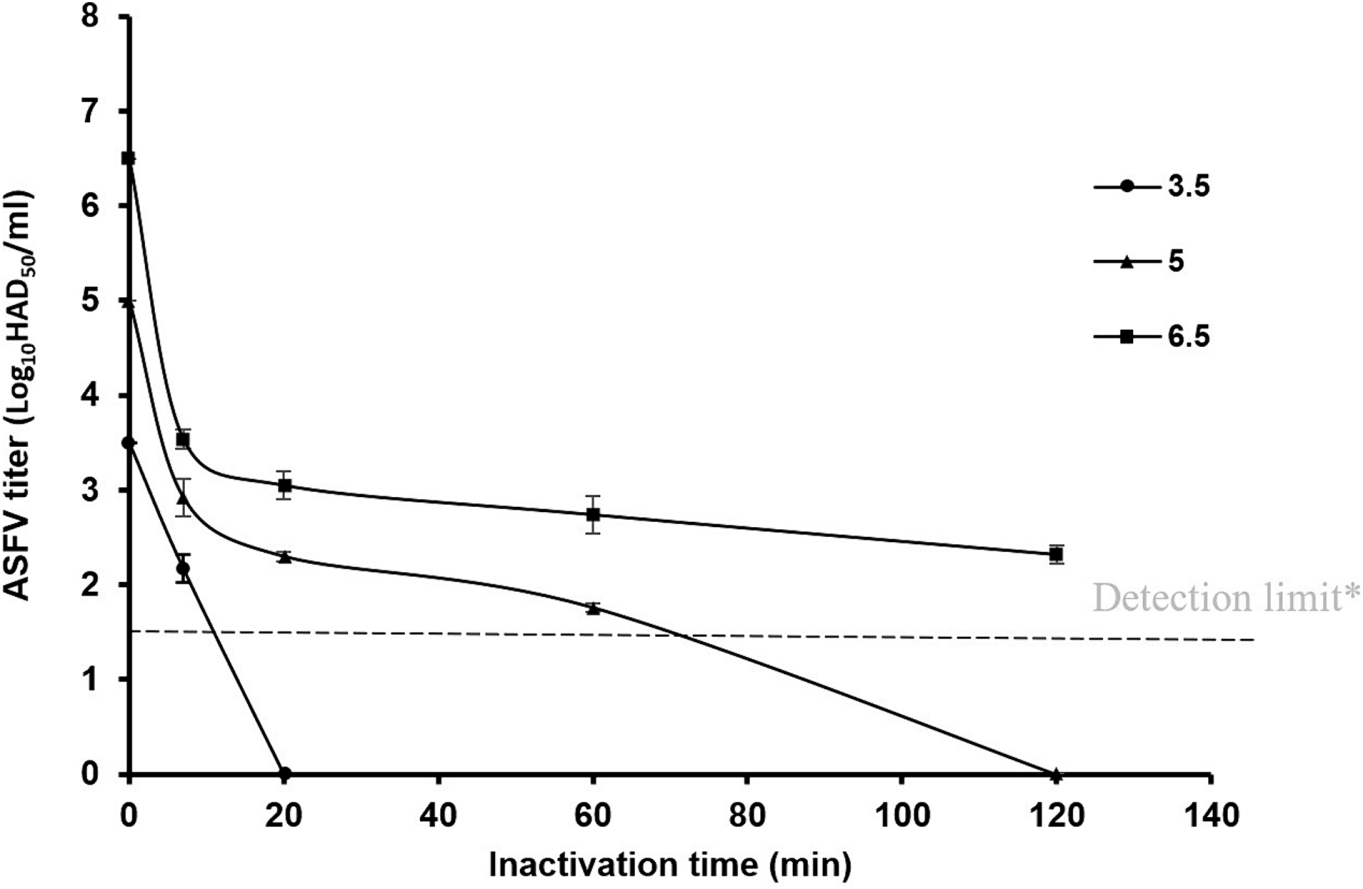
Efficacy of SAFER^®^ at pH 3.2 against ASFV prepared in sterile water at 25°C. *The real-time PCR detection limit for virus detection was 1.5 Log_10_HAD_50_/ml.

ASFV strain VNUA/HY-ASF1/Vietnam/2019, which had virus titers of 6.5, 5, and 3.5 Log_10_ HAD_50_/ml, was used to measure the reduction in ASFV titer over time (0, 7, 20, 60, and 120 min) after treatment with SAFER^®^ at pH 3.2. The results showed that the ASFV virus was undetectable at 3.5 and 5 Log_10_HAD_50_/ml after 20 and 120 min of treatment with SAFER^®^, respectively. However, at a virus titer of 6.5 Log_10_HAD_50_/ml, viral DNA was still detectable after 120 min of SAFER^®^ treatment (Figure 1). Therefore, this ASFV titer was used for subsequent experiments to investigate the effects of temperature and pH on activity. In addition, the maximum incubation time was reduced from 120 to 60 min, as the last 60 min had minimal effect on viral titer.

### 3.2 Antiviral activity of SAFER^®^ at pH 3.2 against ASFV in contaminated fluids at 25°C

**Figure 2:**
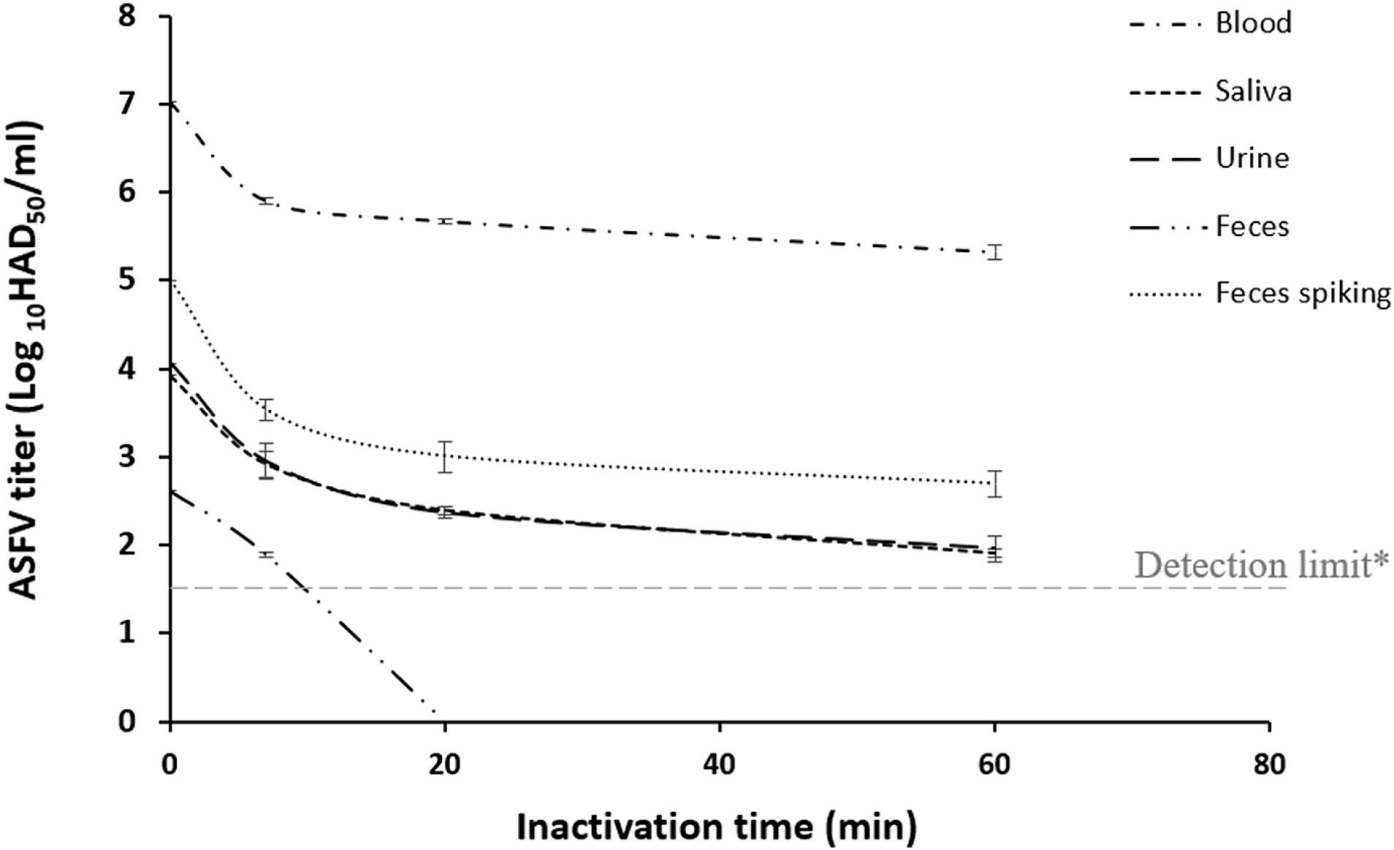
Effectiveness of SAFER^®^ at pH 3.2 to reduce the viral titer of AFSV (Log_10_HAD_50_/ml) in different corporal fluids at 25°C (Sensitivity of real-time PCR for ASFV detection was 1.5 Log_10_HAD_50_/ml).

This study observed that the ASF virus titer in contaminated fluids decreased rapidly within the first 10–20 min after exposure to SAFER^®^. This reduction slowed between 20 min and 1 h after incubation. As shown in Figure 2, after 20 min of exposure to SAFER^®^, virus titers in ASFV-contaminated body fluids such as blood, saliva, urine, and feces were reduced by 1.6, 2.2, 2.0, and 2.2 Log_10_HAD_50_/ml, respectively. Our previous study found that real-time PCR could not detect ASFV titers below 1.5 Log_10_HAD_50_/ml (25). These results demonstrated that the powder form of SAFER^®^ was capable of disinfecting the ASF virus in organic media such as blood, saliva, urine, and feces.

### 3.3 Antiviral activity of SAFER^®^ against ASFV at different temperatures, times, and pH

**Figure 3:**
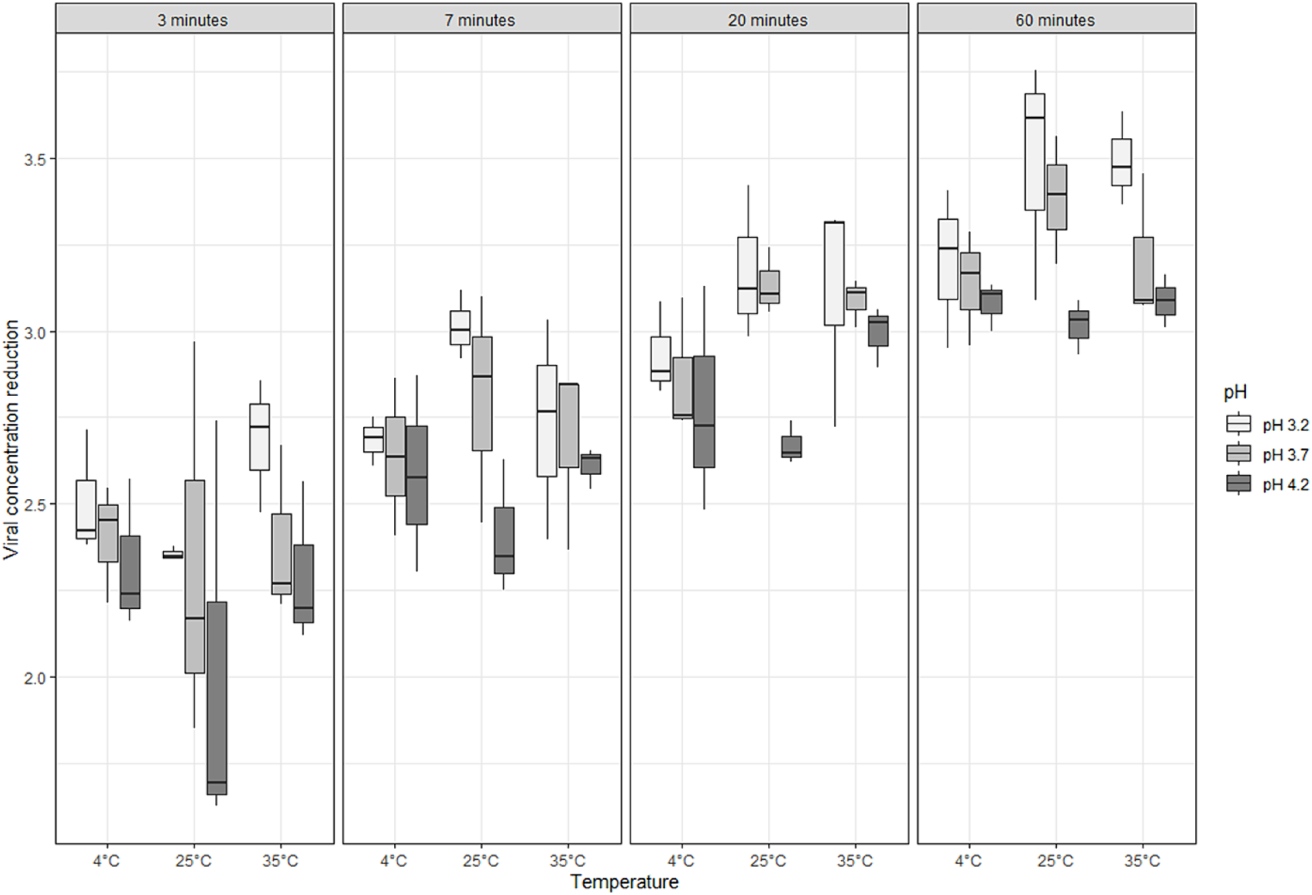
Effect of SAFER^®^ on ASF virus titer reduction (Log_10_HAD_50_/ml) at different combinations of pH, incubation time, and temperature.

The antiviral efficacy of SAFER^®^ against ASFV under different temperatures (4 to 35°C), time (3 to 60 min), and pH (3.2 to 4.2) conditions was evaluated at a baseline viral concentration of 6.5 Log_10_HAD_50_/ml. The virus titer reductions obtained for each factor combination are summarized in Table 2 and shown in the boxplots (Figure 3).

**Table 2:** Reduction of virus titer depending on pH of SAFER, temperature, and incubation time.

Safers	Incubation time (min)	Reduction of ASFV titers (Log_10_HAD_50_/ml)		
		4°C	25°C	35°C
Safer A (pH 3.2)	3	2.51	2.35	2.68
	7	2.68	3.01	2.73
	20	2.93	3.18	3.12
	60	3.20	3.49	3.49
Safer B (pH 3.7)	3	2.40	2.33	2.38
	7	2.64	2.80	2.69
	20	2.86	3.13	3.09
	60	3.14	3.38	3.21
Safer C (pH 4.2)	3	2.32	2.02	2.29
	7	2.58	2.41	2.61
	20	2.78	2.67	2.99
	60	3.08	3.02	3.09

The results showed that ASFV titers were reduced by SAFER^®^ regardless of temperature, pH, and incubation time. Regardless of pH and temperature, all measurements showed an increasing reduction of ASFV over time, with logarithmic reductions of 2.36, 2.68, 2.97, and 3.23 Log_10_HAD_50_/ml for 3, 7, 20, and 60 min, respectively. The reduction in ASFV titer was higher at low pH than at other pH values. At pH 3.2, the average reduction was 2.95 Log_10_HAD_50_/ml; at pH 3.7 and 4.2, it was 2.84 and 2.66 Log_10_HAD_50_/ml, respectively. The effect of temperature on ASFV titer reduction showed fewer clear trends, with ASFV titer reductions of 3.20, 3.49, and 3.49 for 4°C, 25°C, and 35°C, respectively, at pH 3.2 and 60 min of incubation. Higher temperatures (25°C and 35°C) generally resulted in slightly better reduction than 4°C, especially at longer incubation times and lower pH values. The effect of SAFER^®^ on ASFV titers over time showed that a 2 Log_10_ reduction in ASFV was observed rapidly after 3 min of exposure, followed by a more gradual viral log reduction that reached 3 Log_10_ after 20 min at 35°C, 20 min at 25°C, and 60 min at 4°C.

Finally, the significance of each parameter was tested by statistical analysis using the linear model of multifactorial analysis of variance with a 5% risk error (Table 3).

**Table 3:** Statistical analysis a 3-factor ANOVA was applied using R software (R Development Core Team), evaluating the impact of each parameter using the linear model of multi-factors variance analysis with a risk error of 5%.

	SS	df	MS	*F*-value	Pr(>F)
Temperature	0.19	2	0.10	1.88	0.158
pH	1.57	2	0.79	15.23	1.806 e-6***
Incubation time	11.26	3	3.75	72.72	< 2.2 e-16***
Temperature:pH	0.50	4	0.13	2.43	0.053
Residuals	4.96	96	0.05		

***p-value < 0.001.

Statistical analysis showed that temperature alone had no significant effect (*p* > 0.05) on the effectiveness of SAFER^®^, while pH and incubation time had very significant effects. The statistical test showed a tendency for a correlation between temperature and pH with a *p*-value close to significance.

## 4 Discussion

As infectious ASFV is secreted and excreted, it readily contaminates the environment, which serves as a source for the virus. Numerous epidemiological studies have shown that ASFV is easily transmitted through direct or indirect contact with contaminated animal feed or materials such as clothing, footwear, equipment, food waste, and farmers. In 2018, ASF unexpectedly broke out in East Asia and was reported in China and Mongolia, and later in 2019, ASF also occurred in Vietnam (22, 26, 27). The exact origin of this disease in East Asia is unknown and requires further investigation. ASFV DNA has been detected in pig feed, such as dried blood pig feed (26, 28). Several experimental studies have shown that transmission can occur via contaminated feed. However, knowledge of fomite transmission to pigs is relatively limited (29–32).

In this study, the antiviral activity of SAFER^®^ disinfectant powder against ASFV was demonstrated under various physicochemical conditions. At a fixed ambient temperature (25°C) and acidic pH (3.2), the disinfectant powder was able to completely inactivate the ASF virus after 5 min of incubation when present at a moderate concentration (3.5 Log_10_HAD_50_/ml). At higher virus concentrations of 5 Log_10_HAD_50_/ml, 2 h of incubation was required for complete inactivation. The antiviral activity of SAFER^®^ can be explained by its bioactivity, which involves three mechanisms of action: (1) acidification (pH < 3.2), (2) direct antimicrobial action of the active ingredient of thyme essential oil, and (3) the drying action of the clays. An acidic pH is known for its inhibitory effect on pathogen growth, suggesting that bacteria/viruses are sensitive to an acidic pH in the environment. According to the literature, the ASF virus is sensitive to pH below 3.9 (19). In this study, the most effective reduction in viral titer with SAFER^®^ was observed at a pH of 3.2, the lowest pH tested, for all times and temperatures tested. This reduction increases with time, from 2.36 Log_10_HAD_50_/ml at 3 min to 3.23 Log_10_HAD_50_/ml at 60 min. However, it should be noted that even at a pH of 4.2, the highest pH of SAFER^®^ tested, the reduction in viral titer is still significant, albeit less than at a pH of 3.2, which is also the case at a pH of 3.7. A previous study revealed that the virus inactivation rates of sodium bisulfite and ascorbic acid depended on concentration, temperature, and pH value. The influence of pH has also been observed in RNA viruses, where acids compromise the integrity of the protein envelope and the viral genetic material (33). In our study, the efficacy of the product at different pH values appeared to interact with temperature, with better efficacy at pH 3.2 than at higher pH values and higher temperatures (Table 2).

The active ingredient of thyme essential oil is also involved in the antimicrobial properties of the product. In fact, the active ingredient of thyme essential oil has several proven properties, such as antibacterial and antifungal properties (34). Several studies have investigated the antibacterial activity of thyme essential oil extract on both Gram-negative and Gram-positive bacteria, and it was found to be effective on all bacteria tested (35). Other properties, such as insect repellent, antioxidant, anti-inflammatory, cicatrizing, and antiseptic, have also been demonstrated in field studies (36, 37).

The drying effect of clay: unlike most disinfectants on the market, it comes in powder form. SAFER^®^, therefore, does not introduce additional water into the environment. On the contrary, reduces the moisture content of the surface. This is because water is not only a suitable medium for transmitting infectious agents but is also needed for all biochemical reactions of life. However, depending on its availability in the environment, water can be divided into free water (which can react with or bind to other substances) and bound water (unavailable water). Moisture activity measures free water and is a key parameter for pathogen growth. The minimum level of water activity required for cell division depends on the group of microorganisms (38). By reducing water activity, clays are therefore effective inhibitors of pathogen growth. The clays used in the product have a two-stage action: first, they trap free water from the environment, reducing the water activity of the surfaces with which they come into contact. Then, their multilamellar sheet structure increases the contact area between the trapped water and the air, which promotes faster water evaporation. After evaporation, the dried clays regain their ability to trap and evaporate water. In addition, the application in powder form facilitates the visualization of a complete treatment.

In areas affected by ASF or where there is a risk of introduction, disinfectants used for disease control must be effective against ASFV and approved by the official veterinarian. The choice of disinfectants and disinfection procedures should always take into account the nature of the premises, vehicles, and objects to be treated. However, the veterinary service should officially approve the disinfectants and ensure that the conditions for their use are strictly observed. A list of effective chemicals and disinfectants can be found in the literature, but their toxicity limits their use, especially in the presence of animals. Many ASF outbreak investigations have identified biosecurity deficiencies as a critical element in the introduction and spread of the virus. Therefore, it is important to take a holistic biosecurity approach that includes all types of measures to prevent the introduction of new pathogens and reduce their spread on the farm. Rigorous cleaning and disinfection protocols are essential and could combine the use of chemical and non-chemical components, such as antiviral desiccants, to improve and prolong the effectiveness of the process. In summary, the results of this experiment show that SAFER^®^, a non-toxic disinfectant that can be applied in powder form in the presence of animals, is a useful tool for reducing viral loads in the environment, even in the presence of animal fluids. The statistical analysis of the experimental design showed that the efficacy depended mainly on the pH of the product and the exposure time, while the influence of temperature alone was not significant.

## Data availability statement

The original contributions presented in the study are included in the article/Supplementary material, further inquiries can be directed to the corresponding author.

## Ethics statement

The study involving animal participants was reviewed and approved by the Committee on Animal Research and Ethics of the Faculty of Veterinary Medicine, Vietnam National University of Agriculture (approval number: CARE-2021/10; approval date: December 4, 2021).

## Author contributions

EL, MM-M, LP, AM, A-CD, PC, and VL suggested and supervised the experiment ideas. TT, TL, TN, and VL performed the experiments. TT, EL, LP, PC, and VL analyzed the data and prepared the manuscript. All authors contributed to the article and approved the submitted version.
