# Supplementary material for: Antiviral activity of SAFER®, a commercial acidifying desiccant powder, against African swine fever virus
Source: Front Vet Sci. 2024 Aug 20;11:1245569. doi: 10.3389/fvets.2024.1245569 (PMC11369675; doi:10.3389/fvets.2024.1245569)
Supplement: Supplementary file 1 [file Table_1.docx]

**Antiviral Activity of SAFER^®^, a Commercial Acidifying Desiccant Powder, Against African Swine Fever Virus (ASFV)**

Thi Bich Ngoc Trinh^1a^, Elodie Lazenec^2a^, Thi Ngoc Ha Lai^1^, Maria Matard-Mann^2^, Luong Tan Phat^2^, Anne Morvan^2^, Anne-Cecile Delahaye^2^, Pi Nyvall Collén^2^, Thi Lan Nguyen^1^, Van Phan Le^1^*

**Supplementary Table 1**: Experimental design for evaluation of the antiviral activity of SAFER^®^ at pH 3.2 against ASFV isolate at room temperature (25°C) in sterile water

| **STEP** | **FT1**  (SAFER only) | **FT2**  (ASFV isolate only) | **FT3**  (ASFV isolate only) | **FT4**  (ASFV isolate only) | **FT5**  (SAFER, Neutralization broth, and ASFV) | **FT6**  (SAFER, ASFV isolate, and Neutralization broth) | **FT7**  (SAFER, ASFV isolate, and Neutralization broth) | **FT8**  (SAFER, ASFV isolate, and Neutralization broth) | **FT9**  (SAFER, ASFV isolate, and Neutralization broth) | **FT10**  (SAFER, ASFV isolate, and Neutralization broth) | **FT11**  (SAFER, ASFV isolate, and Neutralization broth) |
| --- | --- | --- | --- | --- | --- | --- | --- | --- | --- | --- | --- |
| **1** | Safer 0.3g | - | - | - | Safer 0.3g | Safer 0.3g | Safer 0.3g | Safer 0.3g | Safer 0.3g | Safer 0.3g | Safer 0.3g |
| **2** | 1 ml H_2_O | 1 ml ASFV at 10^3.5^ HAD_50_ | 1 ml ASFV at 10^5^ HAD_50_ | 1 ml ASFV at 10^6.5^ HAD_50_ | 1 ml H_2_O | 1 ml ASFV at 10^3.5^ HAD_50_ | 1 ml ASFV at 10^3.5^ HAD_50_ | 1 ml ASFV at 10^3.5^ HAD_50_ | 1 ml ASFV at 10^3.5^ HAD_50_ | 1 ml ASFV at 10^5^ HAD_50_ | 1 ml ASFV at 10^6.5^ HAD_50_ |
| **Incubation time** | 2h at RT | | | | | 2h at RT | 1h at RT | 20 min at RT | 7 min at RT | 2h at RT | 2h at RT |
| **3** | 1 ml Neutralizing broth (Neutralisant universal; http://www.indicia.fr) | | | | | | | | | | |
| **Contact time** | Mix and allow to rest 10 min at ambient temperature | | | | | | | | | | |
| **4** | 1 ml H_2_O | 1 ml H_2_O | 1 ml H_2_O | 1 ml H_2_O | 1 ml ASFV at 10^3.5^ HAD_50_ | 1 ml H_2_O | 1 ml H_2_O | 1 ml H_2_O | 1 ml H_2_O | 1 ml H_2_O | 1 ml H_2_O |
| **Centrifugation** | Mixing and centrifugation at 4000 rpm for 10 min | | | | | | | | | | |
| **Sampling for PCR** | Transfer 1.5 ml of the supernatant to a clean tube and avoid disturbing the pellet as this may interfere with PCR. Use this sample for the subsequent PCR procedure according to the instructions and volumes of the kit | | | | | | | | | | |
| **PCR** | PCR according to the protocol of the Kit used | | | | | | | | | | |
