# Supplementary material for: Antiviral activity of SAFER®, a commercial acidifying desiccant powder, against African swine fever virus
Source: Front Vet Sci. 2024 Aug 20;11:1245569. doi: 10.3389/fvets.2024.1245569 (PMC11369675; doi:10.3389/fvets.2024.1245569)
Supplement: Supplementary file 2 [file Table_2.docx]

**Antiviral Activity of SAFER^®^, a Commercial Acidifying Desiccant Powder, Against African Swine Fever Virus (ASFV)**

Thi Bich Ngoc Trinh^1a^, Elodie Lazenec^2a^, Thi Ngoc Ha Lai^1^, Maria Matard-Mann^2^, Luong Tan Phat^2^, Anne Morvan^2^, Anne-Cecile Delahaye^2^, Pi Nyvall Collén^2^, Thi Lan Nguyen^1^, Van Phan Le^1^*

**Supplementary Table 2**: Experimental design for evaluation of the antiviral activity of SAFER^®^ at pH 3.2 against ASFV at room temperature (25°C) in contaminated fluids

| **STEPS** | **FT1** | **FT2** | **FT3** | **FT4** | **FT5** | **FT6** | **FT7** | **FT8** | **FT9** | **FT10** |  |
| --- | --- | --- | --- | --- | --- | --- | --- | --- | --- | --- | --- |
|  | ASFV- contaminated blood only | Safer, Neutralizing broth, and contaminated blood | Safer, Neutralizing broth, and ASFV isolate | Neutralizing broth, and ASFV isolate | Safer, contaminated blood, and Neutralizing broth | Safer, contaminated blood, and Neutralizing broth | Safer, contaminated blood, and Neutralizing broth | Safer, ASFV isolate, and Neutralizing broth | Safer, ASFV isolate, and Neutralizing broth | Safer, ASFV isolate, and Neutralizing broth |  |
| **1** | 0.3 mL H20 | Safer 0.3g | Safer 0.3g | 0.3 mL H20 | Safer 0.3g | | | Safer 0.3g | | |  |
| **2** | 0.7 ml contaminated blood | - | - | | 0.7 ml contaminated blood | | | 0.7 ml ASFV isolate at 10^5^ HAD_50_ | | |  |
| **Mixing step** | Mixing to get an homogeneous solution | | | | | | | | | |  |
| **Incubation time** | 0 min at RT | - | - | | 7 min at RT | 20 min at RT | 1 h at RT | 7 min at RT | 20 min at RT | 1 h at RT |  |
| **3** | 2 ml Neutralizing broth | | | | | | | | | |  |
|  |  |  |  |  |  |  |  |  |  |  |  |
| **Contact time** | Mix and allow to rest 10 min at RT | | | | | | | | | |  |
| **4** | - | 0.7 ml contaminated blood | 0.7 ml ASFV isolate at 10^5^ HAD_50_ | 0.7 ml ASFV isolate at 10^5^ HAD_50_ | - | | | - | | |  |
| **Centrifugation** | Mixing and centrifugation at 4000 rpm for 10 min | | | | | | | | | |  |
| **Sampling for PCR** | Transfer 1.5 ml of the supernatant to a clean tube and avoid disturbing the pellet as this may interfere with PCR. Use this sample for the subsequent PCR procedure according to the instructions and volumes of the kit | | | | | | | | | |  |
| **PCR** | PCR according to the protocol of the Kit used | | | | | | | | | |  |

***Note****: This protocol was also used to evaluate the antiviral activity of SAFER® against ASFV in contaminated saliva, urine, feces, and spiked fecal solutions, except that contaminated blood was replaced with another contaminated fluid.*
