# Supplementary material for: Antiviral activity of SAFER®, a commercial acidifying desiccant powder, against African swine fever virus
Source: Front Vet Sci. 2024 Aug 20;11:1245569. doi: 10.3389/fvets.2024.1245569 (PMC11369675; doi:10.3389/fvets.2024.1245569)
Supplement: Supplementary file 3 [file Table_3.docx]

**Antiviral Activity of SAFER^®^, a Commercial Acidifying Desiccant Powder, Against African Swine Fever Virus (ASFV)**

Thi Bich Ngoc Trinh^1a^, Elodie Lazenec^2a^, Thi Ngoc Ha Lai^1^, Maria Matard-Mann^2^, Luong Tan Phat^2^, Anne Morvan^2^, Anne-Cecile Delahaye^2^, Pi Nyvall Collén^2^, Thi Lan Nguyen^1^, Van Phan Le^1^*

**Supplementary Table 3**: Experimental design to evaluate the effects of temperature, pH, and incubation time on the efficacy of Safer®

| **STEPS** | **FT1** | **FT2** | **FT3** | **FT4** | **FT5** | **FT6** | **FT7** | **FT8** | **FT9** | **FT10** | **FT11** | **FT12** | **FT13** | **FT14** | **FT15** |
| --- | --- | --- | --- | --- | --- | --- | --- | --- | --- | --- | --- | --- | --- | --- | --- |
|  | Safer and neutralizing broth | ASFV isolate and neutralizing broth | Safer, neutralizing broth, and ASFV isolate | Safer, ASFV isolate, and neutralizing broth | | | | | | | | | | | |
| **1** | Safer 0.3g | - | Safer 0.3g | 1.5g Safer at pH 3.2 | | | | | | | | | | | |
| **2** | 0.7ml H2O | 0.7 ml ASFV isolate at 10^6.5^ HAD_50_ | - | 3.5 ml ASFV isolate at 10^6.5^ HAD_50_ | | | | 3.5 ml ASFV isolate at 10^6.5^ HAD_50_ | | | | 3.5 ml ASFV isolate at 10^6.5^ HAD_50_ | | | |
| **Temperature** | RT (25 ^0^C) | | | 4 ^0^C | | | | 25 ^0^C | | | | 35 ^0^C | | | |
| **Incubation time** | 1 h | 1 h | 0 min | 3 min | 7 min | 20 min | 1 h | 3 min | 7 min | 20 min | 1 h | 3 min | 7 min | 20 min | 1 h |
| **3** | 1 mL pipetting at each of the 4 timepoints | | | | | | | | | | | | | | |
| **4** | 2 ml Neutralizing broth | | | | | | | | | | | | | | |
| **Contact time** | Mix and allow to rest 10 min | | | | | | | | | | | | | | |
| **5** | - | - | 0.7 ml ASFV isolate at 10^6,5^ HAD_50_ | - | - | - | - | - | - | - | - | - | - | - | - |
| **Centrifugation** | Mixing and centrifugation at 4000 rpm for 10 min | | | | | | | | | | | | | | |
| **Sampling for PCR** | Transfer 1.5 ml of the supernatant to a clean tube and avoid disturbing the pellet as this may interfere with PCR. Use this sample for the subsequent PCR procedure according to the instructions and volumes of the kit | | | | | | | | | | | | | | |
| **PCR** | PCR according to the protocol of the Kit used | | | | | | | | | | | | | | |
